# Assessment of the Aorto-Septal Angle Post-Thoracic Endovascular Aortic Repair through Segmentation and the Semi-Automatic Analysis of Cardiosynchronized Computed Tomography Angiography Images

**DOI:** 10.3390/jcdd11090275

**Published:** 2024-09-04

**Authors:** Marco Magliocco, Michele Conti, Bianca Pane, Marco Canepa, Sara Seitun, Simone Morganti, Giovanni Pratesi, Giovanni Spinella

**Affiliations:** 1 Department of Experimental Medicine, University of Genova, 16132 Genova, Italy; 2 Deparment of Civil Engineering and Architecture, University of Pavia, 27100 Pavia, Italy; 3 3D and Computer Simulation Laboratory, IRCCS Policlinico San Donato, 20097 San Donato Milanese, Italy; 4 Vascular and Endovascular Surgery Unit, IRCCS Ospedale Policlinico San Martino, 16132 Genoa, Italy; 5 Department of Integrated Surgical and Diagnostic Science (DISC), University of Genoa, 16132 Genoa, Italy; 6 Department of Internal Medicine, University of Genoa, 16132 Genoa, Italy; 7 Department of Radiology, IRCCS Ospedale Policlinico San Martino, 16132 Genoa, Italy; 8 Department of Electrical, Computer and Biomedical Engineering, University of Pavia, 27100 Pavia, Italy

**Keywords:** aorto-septal angle, cardiac remodelling, TEVAR, thoracic aorta

## Abstract

The aim of this study was to inviestigate cardiac and arterial remodelling before and after thoracic endovascular aortic repair (TEVAR) by measuring the Aorto-Septal Angle (AoSA) and the geometric characteristics of the aorta. Five patients were prospectively included. Pre- and post-operative cardio-CTA scans were used to create patient-specific 3D models to calculate the AoSA, defined by the intersection of the aortic and left ventricular axes. Additionally, geometric parameters and the inclination of the ascending aorta (AA) were measured. The results demonstrated a variation between pre- and post-operative AoSA in all patients, with an increase in the case of an aneurysmal disease from 112.36° ± 8.21° to 117.16° ± 9.65° (+4.1%, *p* = 0.041) and a decrease in the case of aortic dissection from 113.62° ± 0.96° to 107.83° ± 1.45° (−5.1%). Additionally, an increase in the length of both the outer and inner curvatures of the AA was observed from 102.21 ± 10.17 mm to 105.73 ± 11.2 mm (+ 3.33% *p* = 0.016) and from 55.55 ± 9.53 mm to 58.35 ± 9.96 mm (+4.8%, *p* = 0.04), respectively. This study introduced a new repeatable and reproducible method for assessing the AoSA using cardiac-CTA images. Thoracic stent deployment changes the AoSA, potentially impacting long-term left ventricle hemodynamics.

## 1. Introduction

The Endovascular Treatment of the Thoracic Aorta (TEVAR) is the guideline-recommended treatment for diseases of the descending thoracic aorta [1,2]. The advantages of endovascular treatment have resulted in low mortality and low postoperative complications compared with open repair [3].

Despite this, it is well known that stent-graft deployment increases arterial stiffness and has deleterious effects on cardiac and vascular function [4,5,6] In fact, endovascular treatment has an impact on the aorta, promoting remodelling that can also lead to the onset of complications [7]. 

Much less well-known are the repercussions of endovascular aortic treatment on cardiac structure and function [8]. Among the various alterations, myocardial hypertrophy has been reported by some authors [9]. 

In evaluating this, there are different parameters that can be used, including the angle between the main axis of the ventricle and the aortic axis, which is called the “Aorto-Septal Angle” (AoSA) [10].

The AoSA is a parameter currently used to assess haemodynamic changes in different structural and functional heart disease [11,12]. This parameter has also been used to assess cardiac remodelling after endovascular treatment of the thoracic aorta [13]. 

In current clinical practise, AoSA is evaluated through the direct manual analysis of medical images (ultrasound or CTA), and this measurement is highly operator-dependent and not repeatable [14].

The objective of our study is to describe a methodology for evaluating the AoSA using the segmentation of cardio-synchronised computed tomography angiography (cardiac-CTA), defining a protocol that promotes reproducibility and reliability, and to report aortic geometric parameters before and after TEVAR in five patients.

## 2. Materials and Methods

Patients with thoracic aortic aneurysms who underwent endovascular repair (TEVAR) were enrolled in a prospective imaging study to assess cardiac and aortic deformations following stent deployment.

The enrolment took place after obtaining approval from the regional ethics committee (GR-2019-12371213) and informed consent. All procedures were conducted in accordance with the principles outlined in the Helsinki Declaration, according to the following inclusion criteria: both male and female patients, aged between 60 and 75 years, diagnosed with aortic arch or thoracic aortic aneurysm, and having both pre- and post-operative images available.

The exclusion criteria were previous surgical or endovascular treatments of the abdominal and thoracic aorta, endovascular or hybrid treatments performed on an emergency basis, contraindications to cardiac-CTA examination, medical conditions limiting expected survival to less than 1 year, and the inability to provide informed consent.

For each patient, risk factors, stent graft type, number of stent grafts used, and proximal landing zone were evaluated.

### 2.1. Endovascular Treatment

Briefly, endovascular treatment was performed through a surgical or percutaneous approach of the common femoral artery, a stiff guidewire was placed and, contralaterally, an angiographic catheter was positioned. After the identification of the landing zone, the stent graft was deployed according to preoperative planning.

### 2.2. Image Acquisition

The cardiac-CTA was performed using a dedicated ECG-gated protocol with a second-generation dual source 128 × 2 slices CT scanner (Somatom Definition Flash, Siemens Healthcare, Erlangen, Germany). The protocol is described in detailed in the Supplementary Materials.

Each patient underwent cardiovascular examinations before the procedure and at least 12 months post-procedure, during which a cardio-synchronised CTA scan was performed.

The obtained images were then anonymized and transferred to a workstation for processing.

### 2.3. Geometric Analysis Pipeline

First, an automatic aorta segmentation was performed using the deep learning-based pipeline described in [15].

The obtained model was then complemented with volumes of the coronary arteries, spine, left ventricle, and left atrium, obtained through a semi-automatic segmentation procedure that minimised user interaction, using ITK-SNAP 4.0.2, an open-source software specifically created to segment structures in 3D medical images [16].

The aortic model was used for centreline calculation (from the sinotubular Junction to the iliac branches, excluding the sovra-aortic branches) through a library of tools specifically conceived for the processing of vascular structures, known as the Vascular Model Toolkit library (VMTK) [17]. 

The centreline was then smoothed, resampled, and used for the calculation of geometric parameters: mean diameter, tortuosity, and length using VMTK. Tortuosity index measures the increase in vessel length compared to a straight line connecting the endpoints of the centreline; it was defined as T = L/D − 1, where L is the centreline length and D is the distance between the centreline endpoints.

The diameter was calculated based on the centreline and the surface from which it was derived. Using these two elements, the “vmtkcenterlinesections” function was calculated perpendicular surfaces to the centreline at each point.

These surfaces were considered perfectly circular, and the diameter was derived from their area using the formula D = 2*sqrt(A/pi) through Matlab R2022b (The Mathworks, Natick, MA, USA).

**Figure 1:**
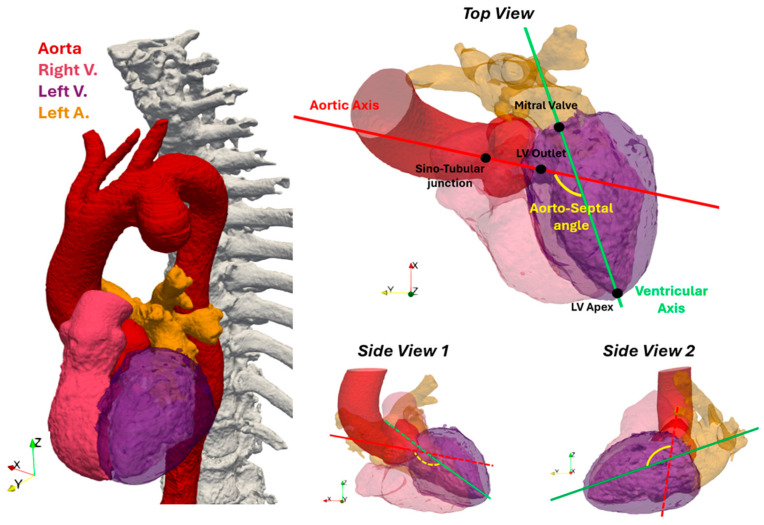
Heart 3D model. On the left side, the complete model is shown, while on the right side, the cardiac model is depicted with three different views, along with the axes used for calculating the aorto-septal angle. The aortic axis is represented in red, and the ventricular axis is in green.

In addition to calculating arterial parameters, it is crucial to understand how cardiac geometry changes following endovascular intervention. This was accomplished by calculating the AoSA, defined as the angle between the longitudinal axis of the left ventricle and the axis of the aorta (Figure 1) [14]. 

**Figure 2:**
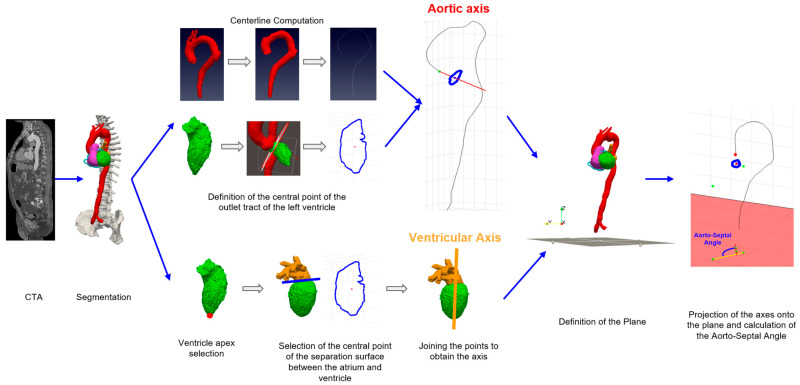
Diagram illustrating the analysis procedure: 3D models were obtained from medical images, which were then used to calculate the aortic centreline and define the aortic axis (in red) and the ventricular axis (in yellow) for the computation of the AoSA (in blue).

As shown in Figure 2, the axis of the aorta was defined by connecting the first point of the centreline (at the sinotubular junction) to the central point of the left ventricular outlet tract. This point was determined by defining a surface within the region of interest based on the segmentation of the ventricle and using a triangulation method to calculate the centroid. 

The longitudinal axis of the ventricle was obtained based on the segmentation of the left atrium and ventricle. The first point was manually selected at the ventricular apex, while the second one was defined by considering the central point of the separating surface between the two structures, where the mitral valve should be located (Figure 2). The method used for defining the axes is similar to that described in [18].

The angle was then calculated using the dot product of the direction vectors of these two axes with respect to a plane parallel to the surface of the heart. The plane was defined using Paraview (Kitware, New York, NY, USA), an open-source application for data analysis and visualisation [19], exported in ‘.csv’ format and imported into Matlab, where the points were projected and the direction vectors were defined (Figure 2).

For each acquisition of each patient, the angle measurement was performed five times; in this way, it was possible to evaluate the intra-observer variability (i.e., the repeatability of the method) by calculating the coefficient of variation, given by the ratio between the standard deviation and the average of the data.

The average of the five measurements was then considered as the angle measurement.

Inter-observer variability was quantified using the Intraclass Correlation Coefficient (ICC). A “Two-Way Random-Effects Model” was chosen. Specifically, two raters assessed all subjects individually and systematically, in order to quantify and generalise the variability to all other raters with similar characteristics [20].

The average percentage variation of the angle was quantified using the following formula:

$$\%=\frac{mean_{pre}-mean_{post}\;}{{mean}_{pre}}\times 100$$

In addition to calculating the AoSA, the inclination of the ascending aorta within its first two centimetres relative to the aortic axis was also determined. For each point along the centreline, direction vectors were computed relative to the initial point, and the dot product with the direction vector of the aortic axis was calculated.

Furthermore, a focus was made on the ascending aorta segment, as it is the section directly connected to the heart and should, therefore, have the greatest impact on the AoSA. In addition to its inclination, its geometric parameters were measured, including the length of the outer and inner curvatures using a method capable of deriving a predefined number of equidistant outer contours positioned on the surface of the vessel, as previously undertaken in our prior work [7]. 

**Figure 3:**
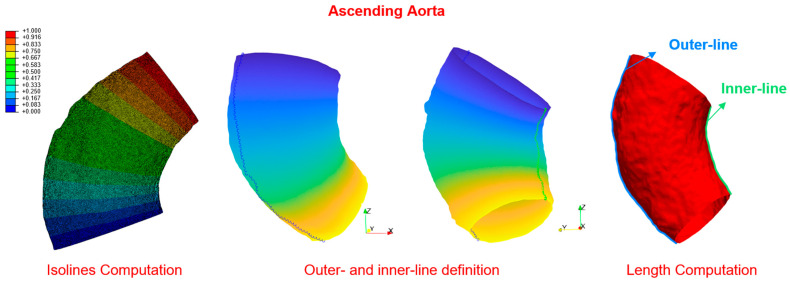
The image shows how to calculate the length of the inner and outer lines of the ascending aorta. In the first image on the left, contour lines are used to represent areas of constant temperature. Next, the points that create the longest path (outer line) and the shortest path (inner line) are identified and connected in order to calculate their lengths.

The method is based on solving a heat conduction problem by setting the vessel’s inlet at a temperature of 1 and the outlet at 0, and on searching for isolines, which are the regions in which the temperature is constant. These isolines are then used to automatically reconstruct the path characterised by the maximum and minimum length (i.e., the length on the outer and inner curvatures), as shown in Figure 3.

### 2.4. Statistical Methods

The variables of interest were reported as mean and standard deviation. Differences between pre- and post-treatment were analysed using a paired sample t-test. The threshold for statistical significance was set at a *p*-value < 0.05. All analyses used GraphPad Prism version 8.0.0 (GraphPad Software, San Diego, CA, USA). Statistical analysis was also performed using the software JMP^®^ Pro 17.0.0.

ICC estimates and their 95% confidence intervals were calculated using the python library “pingouin”, based on a mean-rating (k = 2), absolute agreement, 2-way random-effects model.

## 3. Results

In this study, five patients were prospectively included who underwent endovascular treatment (between October 2021 and January 2022) of the thoracic aorta following the inclusion and exclusion criteria previously described.

Patients’ characteristics, risk factors, and endovascular treatment details are reported in Table 1. Two patients were male and three were female, with an average age of 70 ± 3.4 years. The mean follow-up period was 447.8 ± 72 days.

The intraobserver variability was evaluated via the coefficient of variation (CV), which was always less than 1.5%, with a preoperative mean of 1.17 ± 0.15 and a postoperative mean of 1.02 ± 0.25.

The interobserver variability was instead evaluated through the ICC, and the value obtained was 0.96 with a 95% confidence interval of [0.84; 0.99].

**Figure 4:**
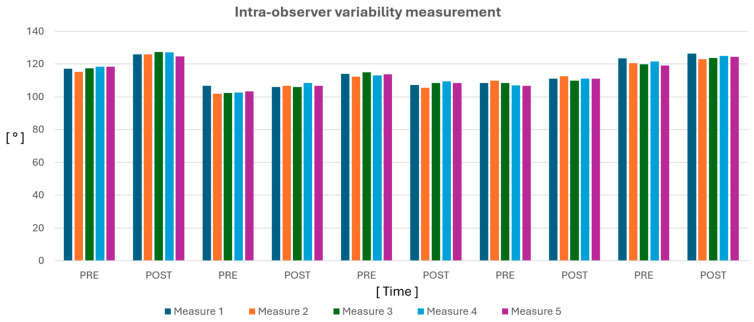
Intra-observer variability measurement.

The measurements carried out by the two observers to evaluate the variability are shown in Table 2 and Figure 4.

The AoSA reported in Table 3 results from the average of five measurements for each patient.

We observed a change in AoSA in all patients after TEVAR. In particular, in all patients who underwent TEVAR for aneurysm, a statistically significant increase in AoSA was observed (*p* = 0.041). On the other hand, a decrease in AoSA was observed only in patients who underwent surgery for aortic dissection. 

Table 4 presents the geometrical characteristics of the ascending aorta. There is a significant variation in the changes in the length of the outer line (*p* = 0.016) and the inner line (*p* = 0.04) between pre- and post-treatment. There are no significant changes in its inclination with respect to the aortic axis (*p* = 0.63). There were no significant changes in its centreline length (*p* = 0.32), in its tortuosity (*p* = 0.76) and in its diameter (*p* = 0.68).

## 4. Discussion

A prospective evaluation to assess changes in AoSA in patients undergoing TEVAR was carried out. 

The first objective of the study was to validate a methodology for measuring the AoSA through the analysis of CT images.

Actually, the AoSA is typically measured using echocardiography, but this method has a few limitations. It is dependent on the transducer’s position and, as a result, is poorly reproducible and highly operator-dependent [14]. In the literature [10,11,12,13], different methods for assessing AoSa using ultrasound have been reported. Instead of the left ventricular axis, the axis defined by the septum or the wall of the right ventricle is used. However, these differences are merely semantic, as the axes are parallel to each other.

The methodology proposed in this article involves generating 3D models from three-dimensional images (cardiac-CTA) to standardise the measurement method. 

Our results demonstrated a favourable coefficient of variation (an index that measures data variability; if it is high, intra-observer variability will be high, resulting in measurements varying significantly from the mean, and conversely, if it is low, there is more consistency among measurements [21,22]). In our case, it was consistently around 1%, thus making this type of analysis reproducible and accurate. Moreover, an ICC of 0.96 suggests a good level of reliability when the analysis is performed by different operators.

To the best of our knowledge, this is the first time a similar method has been proposed for measuring the AoSA, and the consequences of TEVAR on it are also evaluated.

The AoSA is an evaluation that correlates the left ventricle with the ascending aorta. AoSA steep can lead to haemodynamic alterations of the LV that may play a role in the formation of fibrosis of the ventricular outflow tract and the subsequent formation of discrete subaortic stenosis [23]. The formation of fibrosis in the past was considered to be more closely related to genetic conditions; over the years, these assumptions have been modified, and currently it seems that haemodynamic alterations through the interaction with the endocardium may play a role in the formation of fibrosis in the ventricular outflow tract. 

In addition, haemodynamic changes can affect the valve cusps by promoting inflammation and remodelling [24].

Finally, MRI studies have also shown that the haemodynamics of the left ventricle are particularly complex [25].

Recently, some authors, using a non-patient-specific CFD model, have shown how variations in the angle between the ventricle and aorta can alter different parameters. For example, a reduction of about 20 degrees in AoSA can lead to an increase in haemodynamic parameters assessed through simulations, such as wall shear stress variation and mechanical stress alterations, as reported in [26].

Despite there being no statistically significant variations in the parameters (due to the small sample size), what emerges is a change in the AoSA before and after treatment. Specifically, a statistically significant increase is observed in cases of patients with an aneurysm, and a decrease in the angle is seen in the sole case with dissection pathology. For cases where the angle increased, an increase in the left ventricular mass was also observed, from an average value of 61 ± 14.65 g/m^2^ to 68 ± 19.3 g/m^2^. In the case of dissecting pathology, it decreased from 76 to 64 g/m^2^.

In all cases we observed a statistically significant increase in both the inner- and outer-line lengths of the ascending aorta. Although these observations result from an observation of only a few cases, we have in any of these cases observed a homogeneous trend in data in line with what has been observed in larger case studies. For example, Spinella et al. [7] demonstrated that the placement of the stent on the aortic arch can increase the length and curvature of the ascending aorta. Indeed, patients 4 and 5, who had a proximal landing zone in zone 0 and, consequently, the entire aortic arch covered, showed the greatest length variations (outer line +6.3 mm and inner line +2.77 mm for patient 4 and outer line +3.77 mm and inner line +5.81 mm for patient 5). Future studies with a larger number of participants could focus on investigating this aspect and correlating the position of the proximal landing zone and the stent characteristics with the variation in length of the ascending aorta.

Furthermore, it has been demonstrated by some authors [27] that the length of the ascending aorta is closely related to the development of aortic pathologies; in particular, an increase in its length is associated with the development of aneurysmal and dissecting pathologies.

The variations in the inclination of the ascending aorta observed are likely due to the change in configuration resulting from the increase in the length of that arterial segment; as reported by [28], the elongation of the ascending aorta would push this segment downward, causing a decrease in its inclination relative to the axis of the aortic root. In future studies, this aspect will be further investigated and explored in a larger populations to understand whether there is indeed a correlation between changes in the length of the ascending aorta and its inclination.

The provided results may also be useful for the study of endovascular treatment of the ascending aorta, a technique that has not yet been incorporated into routine clinical practise for this vascular segment, but which is currently being investigated through a device called an Endo-Bentall [29]. Our results showed changes in the AoSA, and this could lead to the kinking of coronary artery stents in the case of Endo-Bentall use. 

In addition, as mentioned before, the changes in shape in that area caused by treatment can cause disruptions in blood flow and vascular biomechanics. Therefore, our goal is to compare the geometric analysis results with computational fluid dynamics and biomechanical simulations to observe and understand these changes along with the angle. Currently, studies with a significant number of patients to determine clinical relevance have not been conducted, and they are necessary.

### Limitations

The total number of patients considered in this study is certainly a limitation for drawing clinical conclusions. We also observed variations in a heterogeneous group of patients and different pathologies and proximal landing zones were also considered. In both cases, the aim of the study was the assessment of changes as a result of endovascular treatment. Although these two aspects may have an influence on the results, our results are preliminary and they may be useful in the planning of future studies. However, despite this, it appears evident that the angle undergoes a change one year after endovascular treatment. In the future, the same measurements will be performed on a larger patient pool to reduce variability caused by the small sample size.

One of the issues associated with the measurement method of the AoSA is that the selection of points for defining the aortic axis and the ventricular axis is carried out manually. This problem has been partially mitigated by selecting surfaces and automatically calculating their centroids in the case of the point related to the mitral valve and the one in the left ventricular outlet tract; while the point at the apex of the ventricle is positioned by the user, its position cannot vary much between different measurements due to the elongated shape of the cardiac chamber. In fact, the same observer performing repeated measurements has not shown significant variability in the results obtained.

A potential solution to this issue, to further enhance the analysis by completely eliminating operator-induced variability, would be to automatically select the points, for example by implementing an artificial-intelligence-based method.

## 5. Conclusions

In this study, morphological variations of the aorta and heart following endovascular treatment of the thoracic aorta were investigated through the measurement of the AoSA, the calculation of the ascending aorta inclination, and the determination of geometric parameters of the vessels such as length, diameter and tortuosity.

The results demonstrated an elongation of the ascending aorta and a change in its inclination following endovascular treatment. Furthermore, variations in the AoSA were also observed, thus indicating cardiac remodelling as a result of the intervention.

These are just preliminary results from a small sample size, but the analysis method may be used in the future to study a larger number of patients and, consequently, to draw clinical conclusions. A larger sample will also allow the study of other aspects not considered so far, such as the presence of calcifications and the effect of ageing on angle variations.

Additionally, in addition to the morphological analysis, computational simulations will be performed to describe the variations in the biomechanical and fluid dynamic state of the artery following TEVAR.

## Figures and Tables

**Table 1:** Patients’ characteristics, risk factors and endovascular treatment details.

	Patient 1	Patient 2	Patient 3	Patient 4	Patient 5
Age	73	68	65	72	72
Sex	F	M	F	M	F
Pathology	DTAA	DTAA	TBAD	Aortic Arch Aneurysm	DTAA
Stent Type	Gore	Gore	Bolton	Najuta	Najuta–Gore
Stent Number	1	1	2	1	4
FU Time (days)	511	380	518	413	382
Proximal Landing Zone	4	3	2	0	0
Landing Zone Distance (mm)	160.31	171.06	78.07	74.04	85.03
Stent Coverage Length (mm)	132.3	101	303	169	383
Hypertension	Yes	Yes	Yes	No	Yes
Ischemic Heart Disease	Yes	No	No	No	No
Diabetes	No	Yes	Yes	No	No
Dyslipidemia	Yes	Yes	Yes	Yes	Yes
Smoke	Active	No	Previous	Previous	Previous
COPD	Yes	No	No	Yes	Yes
RI	Yes	No	No	No	No
LV Mass PRE [g/m^2^]	82	56	76	48	58
LV Mass POST [g/m^2^]	80	60	64	54	–

TBAD (type B aortic dissection), COPD (chronic obstructive pulmonary disease), DTAA (descending thoracic aortic aneurysm), RI (renal insufficiency), LV mass (left ventricular mass pre- and post-operative); – represents missing data.

**Table 2:** Inter-observer variability measurement.

	Observer 1		Observer 2	
	PRE	POST	PRE	POST
AS Angle PRE (°)	117.13	128.6	117.25	126.18
AS Angle POST (°)	102.01	106.1	103.43	106.75
AAS (%)	114.05	111.43	113.62	107.83
CV PRE (%)	109.97	107.1	108.05	111.2
CV POST (%)	125.32	125.7	121	124.5

**Table 3:** Aortic features.

	Patient 1	Patient 2	Patient 3	Patient 4	Patient 5
AS Angle PRE (°)	117.25 ± 1.32	103.15 ± 1.35	113.62 ± 0.96	108.05 ± 1.23	121 ± 1.52
AS Angle POST (°)	126.18 ± 1	106.75 ± 0.95	107.83 ± 1.45	111.2 ± 0.99	124.5 ± 1.26
AAS (%)	+7.07	+3.5	−5.1	+2.91	+2.89
CV PRE (%)	1.12	1.35	0.96	1.14	1.26
CV POST (%)	0.8	0.95	1.45	0.89	1

AS angle (aorto-septal angle given by the mean of 5 measurements), CV (coefficient of variation).

**Table 4:** Ascending aorta characteristic.

Patient	AAI (°)	DAA (mm)	TAA (--)	AA CL (mm)	Outer Line Length (mm)	Inner Line Length (mm)
PRE TEVAR						
1	10.95 ± 0.84	37.1	0.07	62.37	90.42	42.81
2	10.54 ± 0.45	32.67	0.21	80.92	108.56	56.75
3	16.61 ± 0.68	31.77	0.09	70.78	95.06	53.24
4	18.6 ± 1.02	34.83	0.14	90.61	115.73	69.50
5	12.55 ± 1.31	40.03	0.09	75.1	101.30	55.46
POST TEVAR						
1	15.74 ± 0.97	37.72	0.07	65.51	94.62	44.8
2	14.57 ± 0.35	31.29	0.17	79.18	110.6	57.38
3	6.52 ± 1.87	32.40	0.11	72.32	96.32	55.63
4	16.86 ± 0.91	34.91	0.17	93.88	122.03	72.27
5	8.44 ± 1.42	40.98	0.1	74.51	105	61.67
∆PRE-POST TEVAR						
1	+4.8	+0.62	0	+3.4	+4.2	+1.99
2	+4	−1.38	−0.04	−1.74	+2.04	+0.63
3	−10.1	+0.63	+0.02	+1.54	+1.26	+2.39
4	−1.73	+0.08	+0.03	+3.27	+6.3	+2.77
5	−4.11	+0.95	+0.01	−1.59	+3.7	+5.81

AAI (ascending aorta inclination, given by the average of 5 measurements), DAA (diameter of the ascending aorta), TAA (tortuosity of the ascending aorta), AA CL (ascending aorta centreline length), outer line length (the length of the external curvature of the ascending aorta), inner line length (the length of the internal curvature of the ascending aorta).

## Data Availability

The data underlying this article will be shared upon reasonable request to the corresponding author.

## Supplementary Materials

The following supporting information can be downloaded at: https://www.mdpi.com/article/10.3390/jcdd11090275/s1, Computed tomography Angiography Protocol.
