# Supplementary material for: Assessment of the Aorto-Septal Angle Post-Thoracic Endovascular Aortic Repair through Segmentation and the Semi-Automatic Analysis of Cardiosynchronized Computed Tomography Angiography Images
Source: J Cardiovasc Dev Dis. 2024 Sep 4;11(9):275. doi: 10.3390/jcdd11090275 (PMC11432616; doi:10.3390/jcdd11090275)
Supplement: Supplementary file 1 [file jcdd-11-00275-s001.zip › jcdd-3160657-supplementary.pdf]

Supplementary material

Computed tomography Angiography Protocol

The CTA protocol of the "Impact of Thoracic Aortic disease endovascular treatment on Cardiac function (ITACA)" study was designed to assess myocardial perfusion, cardiac function and coronary anatomy before and after endovascular treatment of thoracic aortic disease. The protocol is described in detail below.

A non-contrast prospectively electrocardiographic (ECG)-triggered high-pitch spiral acquisition ("Flash" mode) was performed to quantify coronary calcifications by using 120 kVp tube voltage and 40 reference mAs. Secondly, dynamic CT myocardial perfusion scan was performed using alternating table positions (shuttle mode) for complete myocardial coverage, after injecting 40 mL of iodinated contrast agent (Iopamidolo, Iopamiro 370 mgI/mL; Bracco S.p.A., Milan, Italy) at a flow rate of 5 mL/s, followed by a 40 mL saline chaser injected at the same injection rate. The patient's heart was scanned for 30 seconds by using low kV (80 kV), a gantry rotation time of 0.28 second, and a total tube current of 270 mAs per rotation with a scan coverage of 73 mm. Thirdly, a retrospective ECG gating scan of the heart with prospective tube current modulation was performed which allows imaging of the entire cardiac cycle for functional analysis and evaluation of the anatomy of coronary arteries. This was immediately followed by a prospectively ECG-triggered high-pitch spiral acquisition (Flash mode) of the thoracoabdominal aorta and the iliofemoral arteries in the cranio-caudal direction (acquisition duration, ~ 2–3 seconds) by using the same contrast medium bolus. We restricted the total volume of contrast material of this second acquisition (Iopamidolo, Iopamiro 370 mgI/mL; Bracco S.p.A., Milan, Italy) to approximately 70-80 mL by using a triphasic protocol with initial injection of 40-50 mL of undiluted contrast material at 4,5 mL/sec, followed by a 50-mL maintenance bolus of a 60%/40% contrast material–to-saline mixture injected at 4 mL/sec, and 30-40 mL of a saline chaser injected at 4 mL/sec. The cardiac CT examination was automatically started with a delay of 7 seconds after the attenuation of a region of interest in the ascending aorta reached 80 HU. Twenty cardiac phases (each 5% of RR-interval) were reconstructed with a slice thickness of 1.5 mm (increment 1 mm) and multiple systolic and diastolic phases with the least motion artifacts were selected and reconstructed with a thickness of 0.6 mm (increment 0.4 mm) with individually adapted field of view (FOV) for the aortic root and coronary arteries evaluation. The thoracoabdominal aorta was reconstructed with a slice thickness of 1 mm (increment 0.7 mm). Image

reconstruction parameters included a raw-data based iterative reconstruction [kernel I26f, SAFIRE (sinogram-affirmed iterative reconstruction), strength 4] and an effective temporal resolution of 75 ms. For angiographic scans, exposure parameters included 100 kVp tube voltage and algorithm of dose modulation (CAREDose4D, Siemens). Sublingual nitroglycerin to induce coronary vasodilatation and intravenous administration of β blockers for achieving target heart rate were routinely administered if not contraindicated prior to the cardiac examination.
